# Indirubin Increases CD4^+^CD25^+^Foxp3^+^ Regulatory T Cells to Prevent Immune Thrombocytopenia in Mice

**DOI:** 10.1371/journal.pone.0142634

**Published:** 2015-11-16

**Authors:** Aijun Zhang, Bin Ning, Nianzheng Sun, Jianlu Wei, Xiuli Ju

**Affiliations:** 1 Department of Pediatrics, Qilu Hospital, Shandong University, Jinan, China; 2 Department of Orthopaedic, Jinan Central Hospital, Shandong University, Jinan, China; Fudan University, CHINA

## Abstract

Indirubin, a traditional Chinese medicine, is used to treat autoimmune diseases in clinics. However, the effects of indirubin on the immunosuppressive CD4^+^CD25^+^Foxp3^+^ regulatory T cells (Treg) have not been addressed. Thus, we aimed to investigate the effects of indirubin on CD4^+^CD25^+^Treg cells in immune thrombocytopenia (ITP) CBA mice, which were established by immunization with Wistar rat platelets. 50 mg/kg indirubin treatment daily for 4 weeks significantly decreased anti-platelet antibody production and prevented the decrease of platelets caused by immunization in ITP mice. Consistently, indirubin significantly enhanced the percentage and cell number of CD4^+^CD25^+^Foxp3^+^Treg cells in the peripheral blood, spleen and lymph nodes. We also observed a significant increase of the frequency and cell number of CD4^+^CD25^+^Foxp3^+^Treg cells in the thymus upon indirubin treatment. Furthermore, CD4^+^CD25^+^Treg cells from indirubin-treated mice showed similar immunosuppression on T effector cells as compared to those from control mice. Altogether, indirubin ameliorates ITP by enhancing CD4^+^CD25^+^Foxp3^+^Treg cell level with preserving immunosuppressive function.

## Introduction

Immune thrombocytopenia (ITP) is a common autoimmune bleeding disorder characterized by persistent thrombocytopenia in children, caused by the production of anti-platelet autoantibody against platelet membrane glycoproteins which mediates the destruction of platelets in the reticuloendothelial system, especially in the spleen [1]. It has been reported that decreased number and/or function of CD4^+^CD25^+^ Foxp3^+^ regulatory T (Treg) cells, which are crucial for self-tolerance, represent one possible mechanism leading to the development of ITP [2,3].

Indirubin is a traditional Chinese medicine currently used for the treatment of chronic myelocytic leukemia [4] and certain autoimmune conditions and anti-inflammatory therapy [5,6]. Indirubin is a potent cyclin-dependent kinases (CDKs) and glycogen synthetase kinase 3 (GSK-3) inhibitor and suppresses tumor necrosis factor (TNF)-induced NF-κB activation [7]. However, the effects of indirubin on the immunosuppressive CD4^+^CD25^+^ Treg cells have not been addressed. In the present study, we investigated the effect of indirubin on CD4^+^CD25^+^Treg cells in experimental ITP mice.

## Materials and Methods

### Animals

Six-to-eight weeks old female CBA mice were purchased from Model Animal Research Center (Najing, China). Wistar rats were obtained from The Laboratory Animal Center Academy of Military Medical Sciences Genetics (Jinan, China). Mice were maintained for 2 weeks prior experimentation in a specific pathogen-free(SPF) animal facility and were housed in macroisolator cages containing sterilized feed, autoclaved bedding, and water at 20 temperature and 40% humidity condition in the Experimental Animal Centre of Qilu Hospital Shandong University. All the experimental procedures were approved by the Animal Care and Use Committee of Qilu Hospital and conducted under the guidelines for Animal Care and Use of Shandong University, China. All surgery was performed under sodium pentobarbital anesthesia, and all efforts were made to minimize suffering.

### Monoclonal antibodies (mAbs) and reagents

The following mAbs were purchased from BD Biosciences PharMingen (San Diego, CA): FITC-labeled rat anti-mouse CD25 mAb (7D4; IgM), Fluorescein isothiocyanate (FITC)-conjugated anti-mouse CD4 mAb (RM4-5; rat IgG2a), FITC-labeled anti-mouse CD8 mAb (53–6.7; rat IgG2a), phycoerythrin (PE)-labeled rat anti-mouse CD4 mAb(clone GK1.5), PE-labeled anti-mouse CD25 mAb, and PE-labeled anti-mouse CD8α mAb (53–6.7; rat IgG2a). In addition, PE-labeled anti-mouse Foxp3 mAb (FJK-16s) and its staining kit were obtained from eBiosciences (San Diego, CA).

The culture medium used in the present study was RPMI 1640 (Hyclone, Logan, UT) supplemented with 10% heat-inactivated FCS (Hyclone, Logan, UT), 100U/ml penicillin, 100μg/ml streptomycin, 2mM L-glutamine, 10mM HEPES, 1mM sodium pyruvate and 50μM 2-ME (Sigma, St. Louis, MO). DMSO was obtained from Promega Co, Ltd (USA). BSA was purchased from Zhongshan Biotec Co, Ltd (Beijing, China).CD4^+^CD25^+^Treg cells isolation kit was purchased from Miltenyi Biotec(Bergisch-Gladbach, Germany). Mitomycin C (C15H18N4O5) was obtained from Jinmei Co, Ltd. (Beijing, China). [^3^H] thymidine was purchased from China institute of atomic energy (Beijing, China).

### Establishment of ITP animal model

Platelets were isolated from Wistar rat as Musaji described [8]. In brief, blood was collected from jugular vein of Wistar rats with 1/6 volume ACD (citrate-dextrose solution, Sigma-Aldrich, Bornem, Belgium). Platelet-rich supernatant was prepared by successive centrifugations at 10°C for 10 minutes at 200g. Platelets were pelleted from this supernatant by additional centrifugation at 10°C for 12 minutes at 1700g and washed as appropriate. CBA mice were divided into three groups which are control and ITP model group with/without indirubin treatment respectively (n = 10). The two group mice for ITP model were first administered with rat platelets 10^8^ in 0.5 ml saline intraperitoneally, followed by an injection of 0.5×10^8^ platelets weekly. Control mice were injected with saline alone. Indirubin was obtained from Cal Biochem and diluted in the vehicle containing sodium CMC (C-5013 high viscosity, Sigma-Aldrich). Experimental ITP mice received i.p. administration of 50 mg/kg indirubin daily one week before immunization. Pure ITP mice were injected with CMC only.

### Spelnocyte preparation

After mice received indirubin treatment for six weeks, all of the mice were sacrificed by CO_2_ narcosis plus cervical dislocation, spleens was harvested. Single cell suspensions were prepared by grinding the tissues with the plunger of a 5 ml disposable syringe [9]. Cells then were treated with 2 ml hemolysis buffer (17mM Tris-HCl and 140mM NH4Cl, pH7.2) in 1.5–2 min to remove red blood cells. After washed in PBS, the cells were counted and split for FCM staining.

### Immunofluorescence staining and flow cytometry 100μl 1×10^7^/ml

Splenocytes were incubated with 2.4G to block FcRs and then incubated with optimal concentration of fluorochrome-labeled mAbs for 30 minutes at 4°C in dark [10]. Cells were washed three times and resuspended by FCM buffer (PBS with 0.1% BSA and 0.1%NaN_3_). At least ten thousand cells were assayed using a FASCalibur flow cytometry (Becton Dickinson, CA), and data were analyzed with CellQuest software (Becton Dickinson, Mountain View, CA). In some experiments, non-viable cells were excluded using the vital nucleic acid stain propidium iodide (PI).

For the staining of intracellular Foxp3, cells were incubated with Cy-chrome-labeled anti-CD4 and FITC-labeled anti-CD25 mAbs first [11]. Then these cells were fixed after washing and stained with anti-mouse Foxp3 mAb, on the basis of the instruction provided by the company (eBioscience, San Diego, CA).

### The purification of mouse CD4^+^CD25^+^ and CD4^+^CD25^-^ T cells

CD4^+^CD25^+^Treg cells populations were isolated from mouse splenocytes and were enriched using a CD4^+^CD25^+^Treg Cells Isolation Kit with MidiMACS^TM^ Separator on the basis of the instructions offered by manufactory (Miltenyi, Bergisch Gladbach, Germany). The splenocytes were then incubated with a Biotin-antibody cocktail for 20 min at 4°C, laterly with microbead-conjugated anti-biotin mAb (Bio318E7.2) and PE-labeled anti-CD25 mAb. The cell suspension was loaded on a LD column, which is placed in magnetic field of a MACS Separator. The rest cells in the column is the enriched CD4^+^ T cells. In order to the isolation of CD4^+^CD25^+^ cells, the PE-labeled CD25^+^ cells in the enriched CD4^+^ cells fraction were labeled with anti-PE MicroBeads magnetically. These magnetically labeled CD4^+^CD25^+^Treg cells were enriched from all the CD4^+^ cells by MACS sorting. The purity for CD4^+^CD25^+^Treg cells was more than 95% and that for CD4^+^CD25^-^T cells was more than 98% as measured by FCM, then the purified cells were suspended in RPMI 1640 medium completely.

### Immunosuppression assays of CD4^+^CD25^+^Treg cells

CBA splenic CD4^+^CD25^+^Treg cells were isolated from three group mice as described above. 4×10^6^ cells/ml isolated naïve CBA CD4^+^CD25^-^T cells with mitomycin C pre-treated (25 μg/ml at 37°C for 30 min) CBA splenocytes were added into 96-well plates. CD4^+^CD25^+^Treg cells were subsequently added to each well in different ratios to CD4^+^CD25^-^T cells. Cells were cultured with 3μg/ml Con A at 37°C and 5% CO_2_ condition for 4 days [12]. 0.5 μCi [^3^H]thymidine (185GBq/mmol; Atomic Energy Research Establishment, China) was given for the last 16 hours. Cells were harvested with an automatic cell harvester (Tomtec, Toku, Finland). The radioactivity of each sample was assayed in a Liquid Scintillation Analyzer (Beckmon Instruments, America). The counts are expressed as per minute (cpm) of triplicate wells.

### Enzyme-linked immunosorbent assay (ELISA)

ELISA was performed as previously described[13]. Serum levels of INFγ, IL-10 and TGF-β were measured using a commercial ELISA assay kit (BioLegend, San Diego, CA, USA) according to the manufacturer’s instructions.

### Statistical analysis

All data are analyzed by SPSS statistic software and presented as the mean±SD. Student’s unpaired *t* test for comparison of means was used to compare groups. P<0.05 was considered to be statistically significant.

## Results

### Indirubin ameliorates transient thrombocytopenia in mice immunized with Wistar platelets

**Fig 1:**
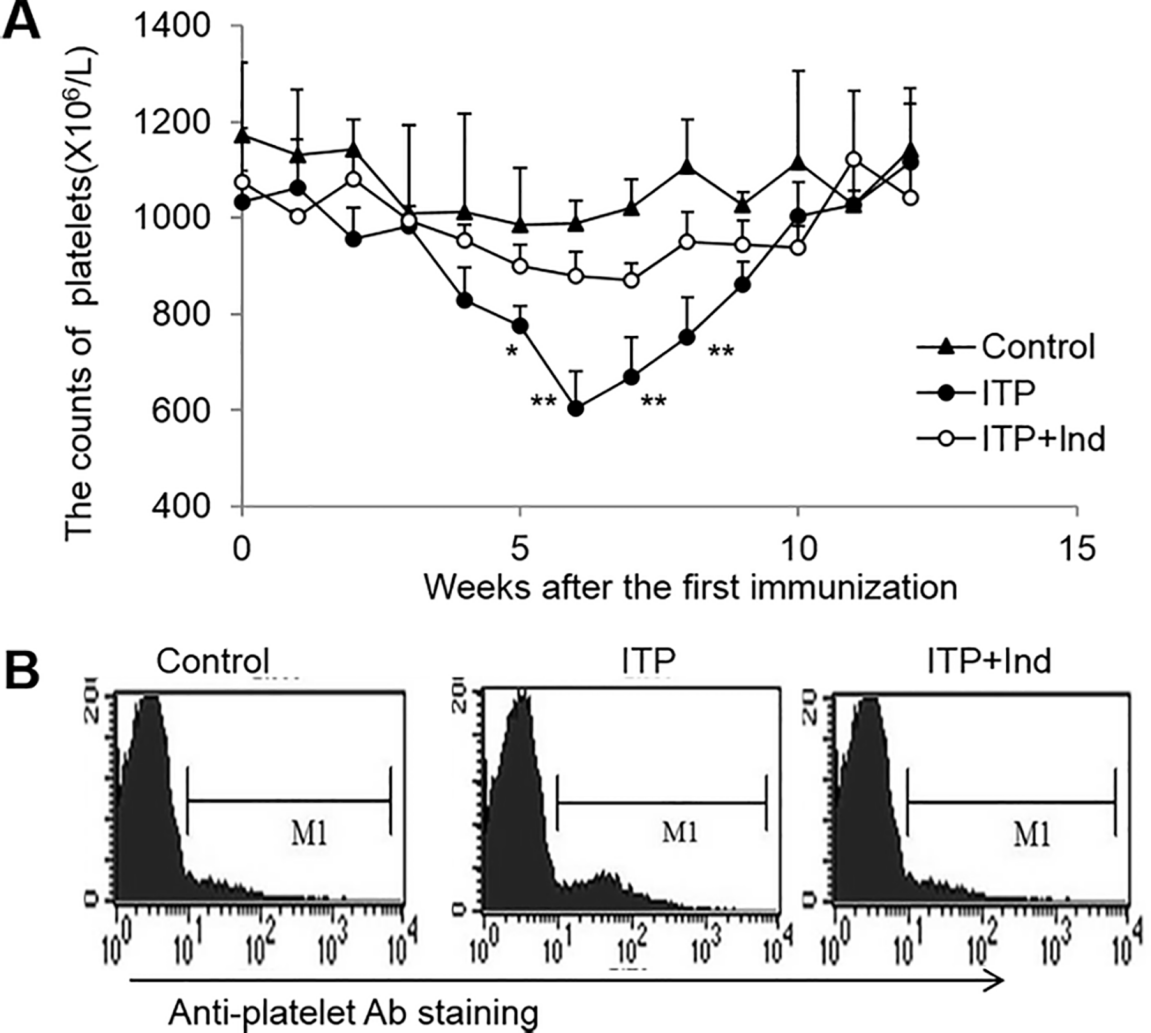
The count of platelets in blood and the staining of anti-platelets antibodies on plates of ITP mice. **A**, The counts of platelets in blood of control mice, ITP mice and indirubin-treated ITP mice were summarized. Values represent mean±SD n = 10 mice of each group. *P<0.05, ** *P*<0.01. **B**, Representative FACS analysis of the staining of anti-platelet antibodies was shown. Representative results are shown from one of three independent experiments performed.

To investigate whether indirubin treatment could prevent the occurrence of ITP caused by immunization with rat platelets in CBA mice, which is a well-established ITP mouse model [14]. CBA mice pretreated or not with 50 mg/kg indirubin daily one week before immunization with rat platelets (10^8^ in 0.5 ml saline) intraperitoneally, followed by 0.5×10^8^ platelets weekly. In this model, the platelet counts gradually decreased and reached to the bottom at the third week after the first immunization. At the sixth week, the platelet number returned to the normal level. However, the decrease of platelet numbers was significantly ameliorated in ITP mice with indirubin pretreatment (*P*<0.01, Fig 1). In line with this observation, the level of anti-platelet autoantibody was also decreased as determined by FCM. Thus, indirubin treatment inhibits the anti-platelet autoantibody production induced by rat platelets administration and subsequently prevents the occurrence of ITP.

### Enhanced percentage and cell number of CD4^+^CD25^+^Treg cells in the periphery of indirubin-treated ITP mice

**Fig 2:**
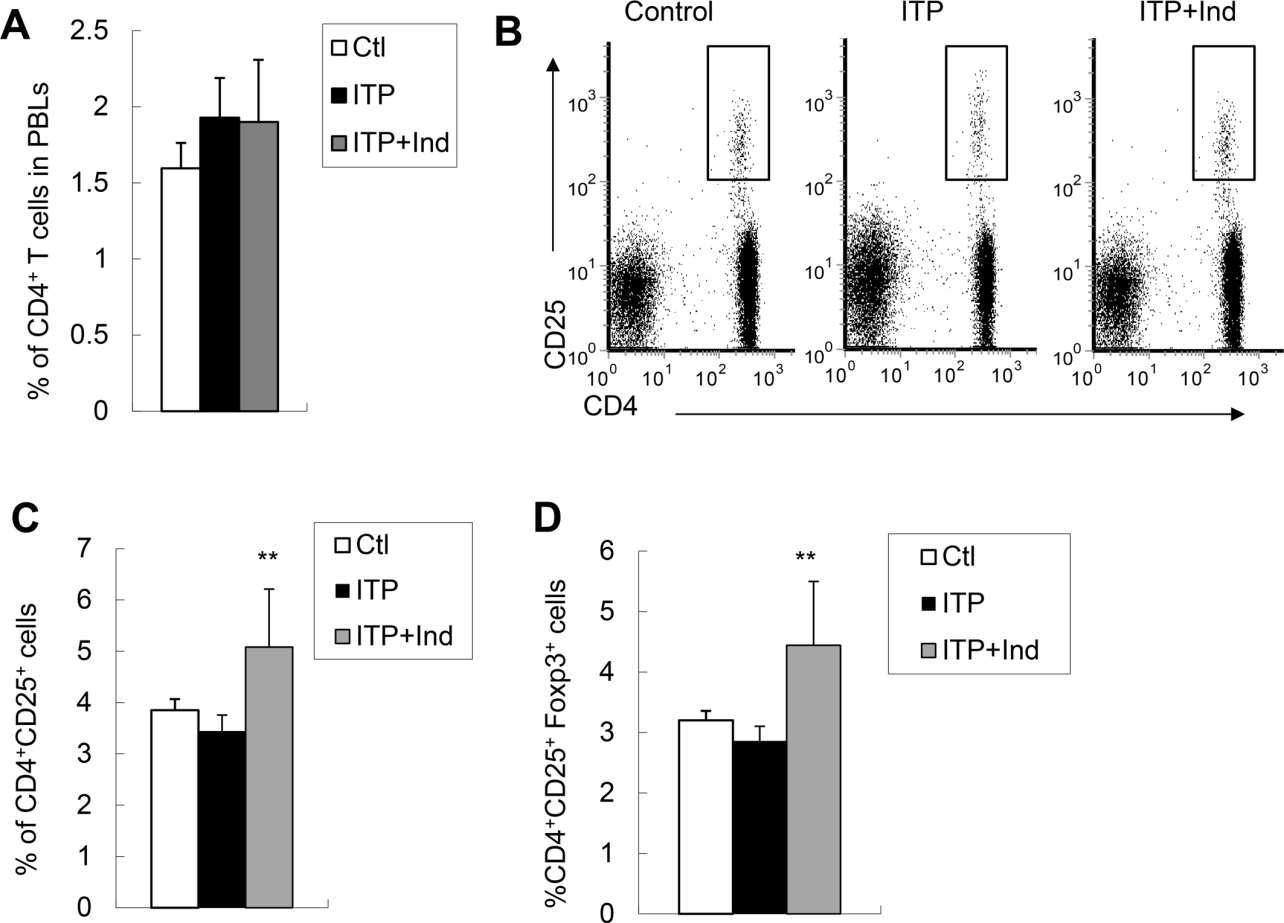
The levels of CD4^+^CD25^+^T cells in the peripheral blood were increased in indirubin-treated ITP mice. **A**, The percentages of CD4^+^T cells in the peripheral blood were unchanged in ITP and indirubin-treated ITP mice. **B**, Representative FACS analysis of CD4^+^CD25^+^ cells in blood was shown. Indirubin significantly increased the percentages of CD4^+^CD25^+^Treg cells (**C**) and CD4^+^CD25^+^Foxp3^+^Treg cells (**D**) in ITP mice. Values represent mean±SD (N = 5). Data are representative of 3 independent experiments. ** P<0.01 compared with ITP mice.

CD4^+^CD25^+^Treg cells play a critical role in immune tolerance and down-regulating immune response [15]. We thus determined the level of CD4^+^CD25^+^Treg cells in peripheral blood of indirubin-treated ITP mice as compared to those in ITP alone or healthy control mice. After 4 weeks of indirubin injection, the percentage of CD4^+^T cells in PBLs was comparable among these mice (Fig 2A). Interestingly, the percentage of CD4^+^CD25^+^Treg cells was significantly increased in indirubin-treated ITP mice as compared to that of ITP mice (*P*<0.01, Fig 2B and 2C). Foxp3 is the key transcription factor for CD4^+^CD25^+^Treg cell development and suppressive function, we thus assessed Foxp3 expression in CD4^+^CD25^+^Treg cells of ITP mice with or without indirubin treatment [16]. Consistent with increased frequency of CD4^+^CD25^+^Treg cells with indirubin treatment, we observed a significant increase of CD4^+^CD25^+^Foxp3^+^Treg cells indirubin-treated ITP mice (P<0.01, Fig 2D). Therefore, indirubin treatment enhanced the level of CD4^+^CD25^+^ Foxp3^+^Treg cells in ITP mice.

**Fig 3:**
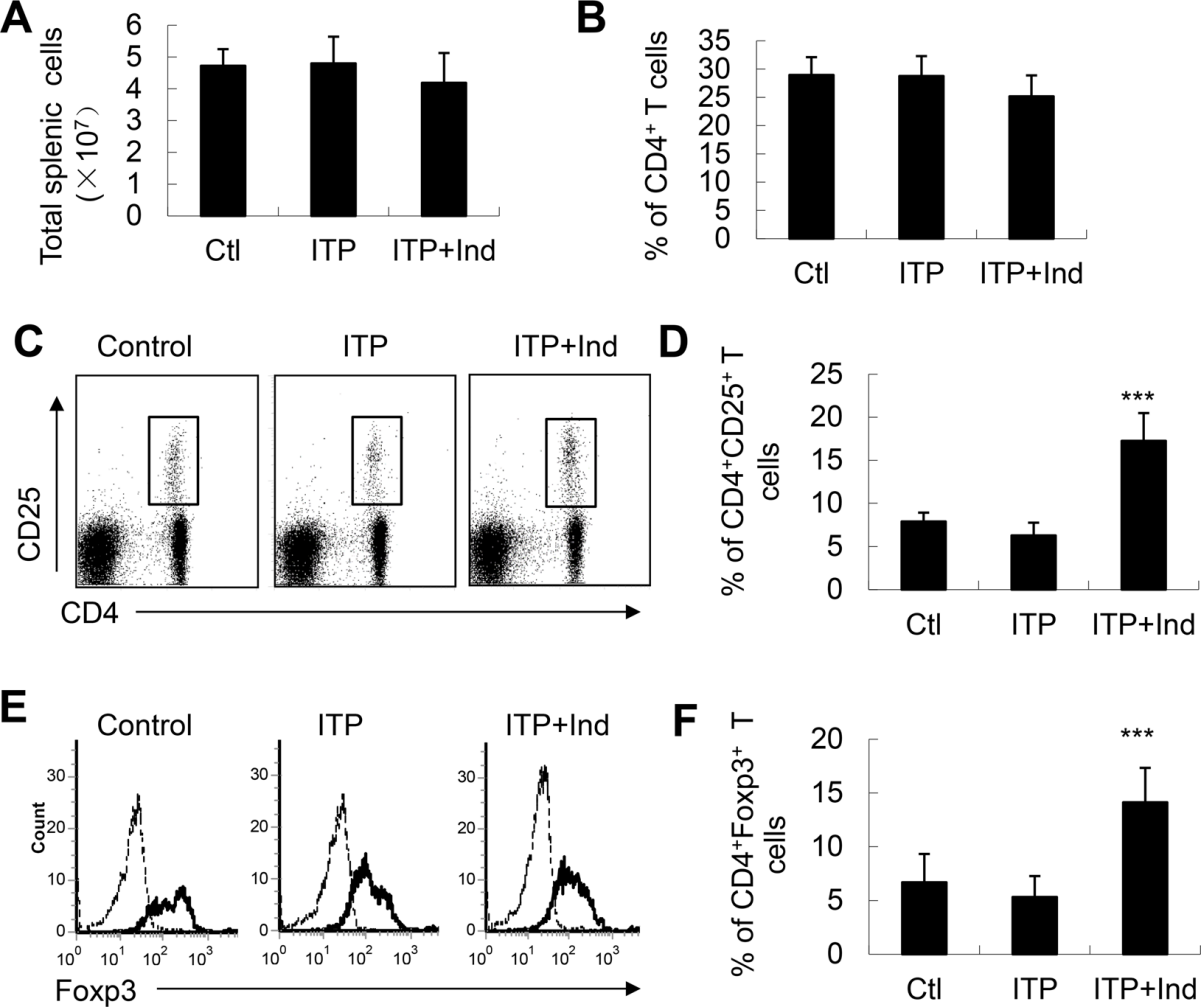
Indirubin increased the levels of CD4^+^CD25^+^T cells in spleens of ITP mice. The total cell number (**A**) and the percentages of CD4^+^T cells (**B**) in the spleens were unchanged in ITP and indirubin-treated ITP mice. **C**, Representative FACS analysis of CD4^+^CD25^+^ cells in spleens was shown. **D**, Indirubin significantly increased the percentages of CD4^+^CD25^+^Treg cells in the spleens of ITP mice. **E**, Representative FACS analysis of Foxp3 expression in gated CD4^+^CD25^+^ cells in spleens was shown **F**, Indirubin significantly increased the percentage of CD4^+^CD25^+^Foxp3^+^Treg cells in ITP mice. Data are representative of 3 independent experiments. *** P<0.001 compared with control or ITP mice.

Consistent with the peripheral blood, the percentage and total cell number of CD4^+^T cells in spleen had no significant changes in ITP mice (Fig 3A). The percentages of CD4^+^CD25^+^Treg cells and CD4^+^CD25^+^Foxp3^+^Treg cells in spleen of ITP mice were remarkably increased after indirubin treatment, compared to ITP mice (*P*<0.001, Fig 3C and 3D). Foxp3, a unique transcription factor for CD4^+^CD25^+^Treg cells, is predominantly expressed in the CD4^+^CD25^+^Treg cells [17]. Therefore, the expression of Foxp3 in CD4^+^CD25^+^T cells in these mice was assessed. As shown in Fig 3, the percentage of CD4^+^CD25^+^Foxp3^+^Treg cells was increased after indirubin treatment (*P*<0.001, Fig 3E and 3F). Moreover, the mean fluorescence intensity of Foxp3 on a per cell basis was also similar among all the groups (data not shown). The absolute cell number of CD4^+^CD25^+^Foxp3^+^Treg cells in spleen was significantly increased compare to those of untreated ITP mice (data not shown). On the other hand, the percentage and absolute cell number of CD4^+^CD25^+^Foxp3^+^Treg cells in peripheral lymph nodes of indirubin-treated ITP mice were significantly higher than those of untreated ITP mice (data not shown).

Furthermore, we determined the phenotype of CD4^+^CD25^+^T cells in ITP mice with or without indirubin treatment. Both CD4^+^CD25^-^T cells and CD4^+^CD25^+^T cells in indirubin-treated ITP mice showed similar expressions of CD44, CD45, GITR and CD152 as those in ITP mice (data not shown), indicating the surface phenotypes were unaltered after indirubin treatment.

### Preserved immunosuppressive and anti-inflammatory ability of CD4^+^CD25^+^Treg cells in indirubin-treated ITP mice

**Fig 4:**
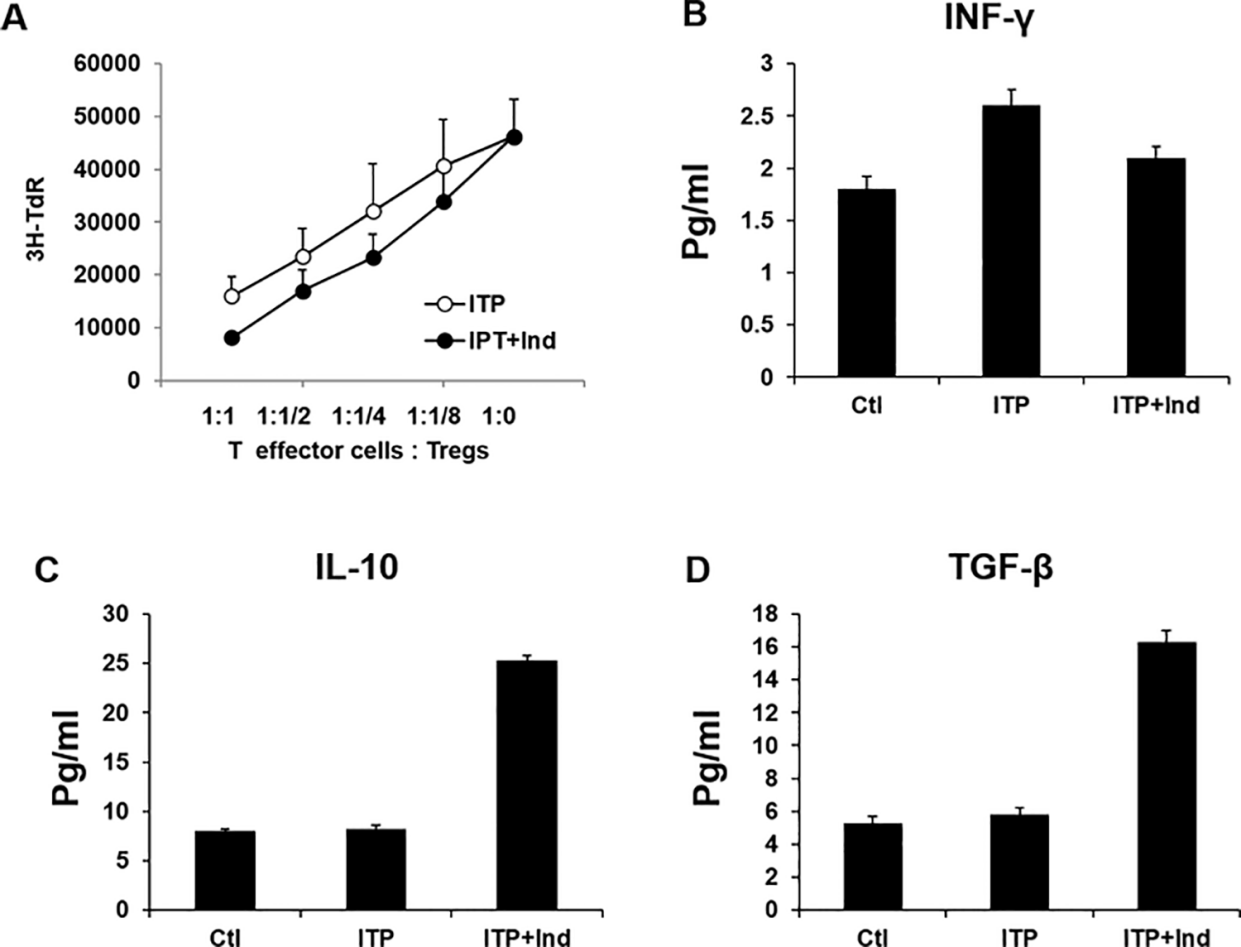
The immunosuppressive and anti-inflammatory function of CD4^+^CD25^+^T cells of indirubin-treated ITP mice was unchanged. (A) The immunosuppressive function of CD4^+^CD25^+^Treg cells isolated from either ITP or indirubin-treated ITP mice were detected as described in materials and methods.(B) INFγ expression of CD4+T cells in absence or presence of indirubin, assayed by ELISA. (C-D) Sera level of IL-10 and TGF-β, assayed by ELISA. Data are representative of 3 independent experiments. *P<0.05, ** P<0.001 compared with control or ITP mice.

To further assess whether indirubin alters the suppressive function of CD4^+^CD25^+^Foxp3^+^Treg cells in ITP mice, we thus compared the immunosuppressive effects of CD4^+^CD25^+^Treg cells sorted by bead separation from ITP mice treated with or without indirubin using an in vitro immunosuppressive assay [18]. Naive CD4^+^CD25^-^T cells were used as responder cells as described in the materials and methods. As shown in Fig 4, both CD4^+^CD25^+^Treg cells from mice treated with indirubin or not showed inhibition on the proliferation of CD4^+^CD25^−^T cells induced by mitogen ConA in a dose-dependent manner (Fig 4A). No detectable difference between CD4^+^CD25^+^Treg cells from indirubin-treated ITP mice and un-treated ITP mice was observed (p>0.05, Fig 4A). Interferon gamma (IFNγ), or type II interferon, is a cytokine that is critical for innate and adaptive immunity against infections. IFNγ is an important activator of macrophages and inducer of Class II major histocompatibility complex (MHC) molecule expression. Aberrant IFNγ expression is associated with a number of auto-inflammatory and autoimmune diseases. The importance of IFNγ in the immune system stems in part from its ability to inhibit viral replication directly, and most importantly from its immunostimulatory and immunomodulatory effects. IFNγ is produced predominantly by natural killer (NK) and natural killer T (NKT) cells as part of the innate immune response, and by CD4 Th1 and CD8 cytotoxic T lymphocyte (CTL) effector T cells once antigen-specific immunity develops. To further determine the anti-inflammatory effect of indirubin, we isolated primary CD4+T cells from ITP mice, followed by incubation in absence or presence of indirubin for 3 days. As demonstrated in Fig 4B, indirubin inhibits the expression of IFNγ *in vitro*. CD4(+)CD25(+)Foxp3(+) regulatory cells (Tregs) are a special lineage of cells central in the maintenance of immune homeostasis, and are targeted for human immunotherapy. They are conventionally associated with the production of classical anti-inflammatory cytokines such as IL-10 and TGF-β, consistent to their anti-inflammatory functions. To investigate CD4(+)CD25(+)Foxp3(+) relative cytokines, we isolated sera from mice and performed ELISA assay. As indicated in Fig 4D & 4E. indirubin-treated group exhibited a higher level of IL-10 and TGF-β compared to control group, indicating indirubin preserved anti-inflammatory effect. These data suggested that indirubin increased CD4^+^CD25^+^Treg cells without altering their immunosuppressive function and preserved an anti-inflammatory effect.

### Indirubin increased naturally occurring CD4^+^CD25^+^Treg cells in the thymi of ITP mice

**Fig 5:**
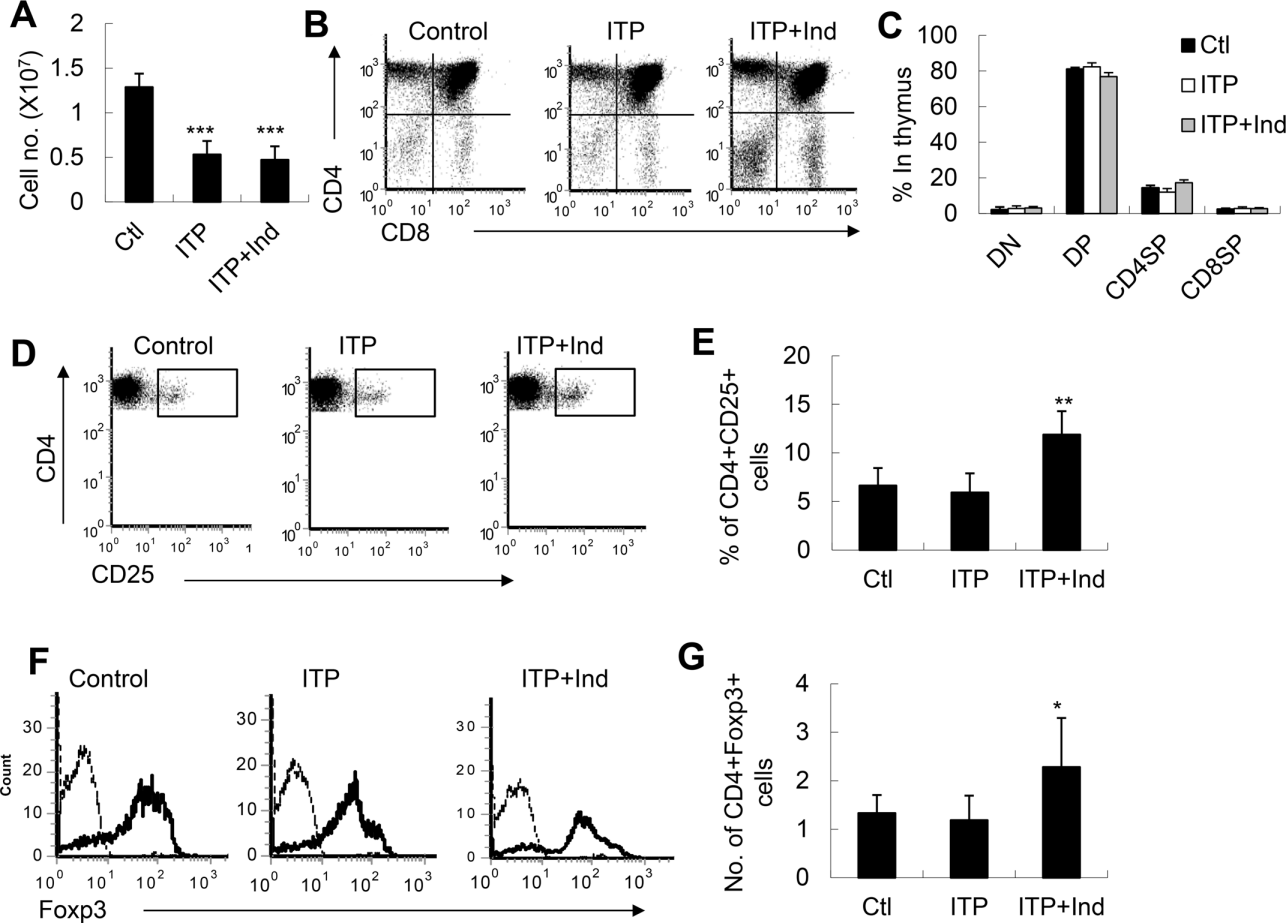
Indirubin increased the levels of CD4^+^CD25^+^Treg cells in the thymi of ITP mice. **A**, The total cell number of thymocytes were decreased in ITP and indirubin-treated ITP mice. **B**, Representative FACS analysis of thymocyte subsets was shown. **C**, The percentages of thymocyte subsets were unchanged in ITP and indirubin-treated ITP mice. **D**, Representative FACS analysis of Foxp3 expression in gated CD4^+^CD25^+^ cells was shown. **E**, Indirubin significantly increased the percentages of CD4^+^CD25^+^Treg cells in the thymi of ITP mice. **F**, Representative FACS analysis of Foxp3 expression in gated CD4^+^CD25^+^ cells was shown. **G**, Indirubin significantly increased the cell number of CD4^+^Foxp3^+^Treg cells in ITP mice. Data (mean±SD, N = 5) are representative of 3 independent experiments. *** P<0.001 compared with control or ITP mice.

CD4^+^CD25^+^Treg cells are mainly derived from thymus and output to the periphery. These Treg cells are called naturally occurring CD4^+^CD25^+^Treg cells (nTreg cells). Therefore, we further detected the level of nTreg cells in the thymi of indirubin treated ITP mice. As shown in Fig 5, the total thymocyte number was markedly decreased in ITP mice regardless of treatment with indirubin or not (Fig 5A). However, the percentages of CD4^+^CD8^-^ (CD4SP), CD4^-^CD8^+^ (CD8SP), CD4^-^CD8^-^ (DN) and CD4^+^CD8^+^T (DP) cells in the thymus were identical among these mice, indicating that ITP impairs thymocyte subset homeostasis equally. Interestingly, the percentages and total cell numbers of CD4^+^CD8^-^CD25^+^ cells and CD4^+^CD8^-^Foxp3^+^nTreg cells were significantly increased in indirubin-treated ITP mice (P<0.01, Fig 5D–5G). Therefore, indirubin might selectively increase nTreg cells in the thymus in ITP mice.

## Discussion

ITP is an autoimmune bleeding disorder caused by the production of anti-platelet autoantibodies that bind to platelet membrane glycoproteins or by activation of platelet-specific cytotoxic T cells and NK cells mediating the destruction of platelets [19,20]. Nowadays, more and more patients tend to develop chronic disease progression and are liable to long-term glycocorticoid treatment or immunosuppressive reagents administration or even splenotomy, which have many side effects and could not cure the patients and are even dangerous for life [21]. Indirubin is a traditional Chinese medicine used in clinics for the treatment of chronic myelocytic leukemia. Indirubin has the ability to inhibit numerous important protein kinases including CDKs [7]. It has been demonstrated that indirubin derivatives have the potent anti-proliferative and inducing apoptosis activity in various human cancer cells [22]. Indirubin and its derivatives have also been reported to exert an anti-inflammatory effect mediated by suppression of the release of cytokine, including IFN-γ and IL-6. Treatment with indirubin derivatives also showed potent anti-proliferative activity in various human cancer cells and induced the activation of caspase-7 followed by apoptosis in RK3E-ras cells [23]. In addition, it might be useful in treating Alzheimer’s disease or diabetes, too [24]. In the present study, we demonstrated that indirubin has the ability to increase immunosuppressive CD4^+^CD25^+^Treg cells. Thus, the treatment with indirubin may be beneficial to induce immune tolerance to self and allo-graft antigens.

CD4^+^CD25^+^ Treg cells are the key players for negatively regulating immune responses [25,26]. Foxp3 is predominantly expressed in the CD4^+^CD25^+^Treg cells and recognized as the key transcription factor for Treg cell development and function [27,28]. Induced expression of Foxp3 by TGF-β in CD4^+^CD25^-^T cells can convert these cells into Treg cells that displayed similar phenotype and function as naturally occurring CD4^+^CD25^+^Treg cells [27]. A significant increase in the percentage of CD4^+^CD25^+^Foxp3^+^Treg cells was observed in the periphery of ITP mice after indirubin treatment. In the *in vitro* immunosuppressive assay, CD4^+^CD25^+^Treg cells from indirubin-treated mice showed comparable immunosuppressive ability with control CD4^+^CD25^+^Treg cells. Altogether, these data indicated that CD4^+^CD25^+^Treg cells are more resistant to inhibition of indirubin as compared with CD4^+^CD25^-^T cells. The selective enhancement of CD4^+^CD25^+^Treg cells by indirubin could efficiently suppress immune response and rescue the imbalance of T cell subsets in ITP mice, which provides a new strategy for ITP treatment.

Interestingly, indirubin significantly increased the percentage and cell number of CD4^+^CD25^+^Treg cells in the thymi of ITP mice. These data suggest that indirubin promotes nTreg cell development in the thymus. Though the mechanisms for this effect were unclear at this moment, we speculate that indirubin might has a therapeutic effect in ITP, at least, partially by promoting the differentiation of CD4^+^CD25^-^ Treg precursor cells into CD4^+^CD25^+^Treg cells. In this regard, the enhanced nTreg development by indirubin likely improves the peripheral immune tolerance. The ability of indirubin to significantly increase thymus derived and peripheral CD4^+^CD25^+^Treg cells makes it attractive as a favored medication for autoimmune diseases or induction of transplantation tolerance.

## Supporting Information

S1 File (PDF)

## Abbreviations

ITP: immune thrombocytopenia
CTLA4: cytotoxic T-lymphocyte–associated protein 4
FCM: flow cytometry
FITC: fluorescein isothiocyanate
Foxp3: forkhead box protein 3
GITR: glucocorticoid-induced tumor necrosis factor receptor
MLR: mixed leukocyte reactions
PBMCs: peripheral blood mononuclear cells
PE: phycoerythrin
PI: propidium iodide
Treg: regulatory T cells
