# Supplementary material for: Indirubin Increases CD4+CD25+Foxp3+ Regulatory T Cells to Prevent Immune Thrombocytopenia in Mice
Source: PLoS One. 2015 Nov 16;10(11):e0142634. doi: 10.1371/journal.pone.0142634 (PMC4646632; doi:10.1371/journal.pone.0142634)
Supplement: S1 File — (PDF) [file pone.0142634.s001.pdf]

山东大学齐鲁医院科研伦理委员会

批　　准　　书

批准号：KYLL-2013(KS)-173

项目名称：靛玉红诱导免疫性血小板减少症小鼠模型免疫耐受的机制研究

英文名称：The Study of Immune Tolerance Induced by Indirubin in Immune Thrombocytopenia mice

项目负责人：鞠秀丽（Xiuli Ju）　　**职称：**主任医师　　**联系电话：**18560086212

负责研究单位：山东大学齐鲁医院

合作研究单位：无

研究起止时间：2013 年 1 月-2014 年 12 月

拟申报项目类别及资助金额（或在研项目资金来源及金额）：

国家自然基金面上项目、20 万元

评审意见：

研究项目

“靛玉红诱导免疫性血小板减少症小鼠模型免疫耐受的机制研究”

经伦理委员会审查：

研究者的资格、经验符合试验要求；研究方案符合科学性和伦理原则的要求；获得知情同意的方法适当；受试者可能遭受的风险程度与研究预期的受益相比合适。

同意开展该项目的研究。

山东大学齐鲁医院科研伦理委员会

主任委员：

2013 **年　2 月 10 日**
